# A database of thermally activated delayed fluorescent molecules auto-generated from scientific literature with ChemDataExtractor

**DOI:** 10.1038/s41597-023-02897-3

**Published:** 2024-01-17

**Authors:** Dingyun Huang, Jacqueline M. Cole

**Affiliations:** 1 Cavendish Laboratory, University of Cambridge, J. J. Thomson Avenue, Cambridge, CB3 0HE UK; 2 ISIS Neutron and Muon Source, Rutherford Appleton Laboratory, Harwell Science and Innovation Campus, Didcot, Oxfordshire OX11 0QX UK

**Keywords:** Biomedical materials, Lasers, LEDs and light sources, Cheminformatics, Single-molecule fluorescence, Optical materials

## Abstract

A database of thermally activated delayed fluorescent (TADF) molecules was automatically generated from the scientific literature. It consists of 25,482 data records with an overall precision of 82%. Among these, 5,349 records have chemical names in the form of SMILES strings which are represented with 91% accuracy; these are grouped in a subsidiary database. Each data record contains one of the following four properties: maximum emission wavelength (*λ*_EM_), photoluminescence quantum yield (PLQY), singlet-triplet energy splitting (Δ*E*_ST_), and delayed lifetime (*τ*_D_). The databases were created through text mining using ChemDataExtractor, a chemistry-aware natural-language-processing toolkit, which has been adapted for TADF research. The text-mined corpus consisted of 2,733 papers from the Royal Society of Chemistry and Elsevier. To the best of our knowledge, these databases are the first databases that have been auto-generated for TADF molecules from existing publications. The databases have been publicly released for experimental and computational applications in the TADF research field.

## Background & Summary

Thermally activated delayed fluorescence (TADF) has become an area of increased interest over the past ten years due to its applications in organic light-emitting diodes (OLED)s. TADF materials can harvest light from non-emissive triplet (T1) states by converting them into emissive singlet (S1) states through thermally induced reverse intersystem crossing (RISC), thus potentially reaching a theoretical internal quantum efficiency (IQE) of 100%^1^. TADF-based OLEDs overcome the intrinsic 25% IQE limit for conventional pure fluorescent OLEDs^2^ and the 75% limit for pure phosphorescent OLEDs^3^. Compared to metal-organic complexes, organic TADF-based materials also do not contain expensive and potentially toxic heavy metal ions^2^. As a key class of the third-generation OLEDs, the discovery and development of better TADF-based light-harvesting molecules for OLED applications is imperative.

Conventional trial-and-error synthetic processes, which take years of effort, have often been the bottleneck of materials discovery. Over the last decade, data-driven design-to-device pipelines have emerged to target the discovery of new functional materials^4^. This field has developed significantly and rapidly in line with the recent advances in supercomputing and machine learning (ML) capabilities. High-throughput (HT) calculations have led to the collation of several large simulated databases^5,6^, which has facilitated downstream materials discoveries^7^. Successful applications of such methods have also been observed in the TADF field^8,9^. In contrast to theoretically simulated information, databases containing materials and properties can also be automatically generated from previously reported scientific literature. Their data are usually very reliable given that they often based on experimental results and have been vetted by peer review. The chemistry-aware natural-language-processing (NLP) toolkit ChemDataExtractor^10–12^ provides a means of this automatic data extraction from the scientific literature^13–21^. The thus obtained databases are characterized by consistent computer-readable structures and can subsequently be used for the analysis of structure-property relationships (SPRs) which provide the patterns required for predicting and thence validating the discovery of new materials for a given application^20,21^.

To the best of our knowledge, there is currently no consistently structured or auto-generated database for existing TADF molecules. To address this shortcoming, ChemDataExtractor v2.1^10–12^ was used to extract data from 2,733 scientific articles about materials and four TADF-relevant properties, i.e., the maximum emission wavelength *λ*_EM_, the photoluminescence quantum yield (PLQY), the singlet-triplet energy splitting Δ*E*_ST_, and the delayed lifetime *τ*_D_. This work endowed ChemDataExtractor v2.1 with a new compound-searching system that is specifically geared for text mining in the TADF field of research. The search yielded 25,482 data records with 82% overall precision. Moreover, a subsidiary database comprising 5,349 property records was also prepared, whose chemical names are represented by additional simplified molecular-input line-entry system (SMILES) strings. Details of the data-extraction pipeline for this work are provided in the *Methods* section, and the performance and reliability of the database are analysed in the *Technical Validation* section.

## Methods

### Article retrieval

The first stage of the automatic database-generation pipeline is to search and retrieve papers from scientific publishers online. In total, 2,733 papers were retrieved under copyright permission, i.e., 1,269 from the Royal Society of Chemistry (RSC) and 1,464 from Elsevier. ChemDataExtractor 2.1 provides bespoke web scraping modules for publishers to perform searches and download articles. The codes build on Elsevier’s application programming interface (API) to perform online requests, which requires a valid API key. The RSC had not provided its own API by the time of the retrieval, and ChemDataExtractor used “urlib3” and “requests” libraries to query the RSC website for paper search and retrieval. The Elsevier papers were received in ‘Extensible Markup Language (XML)’ format, while the RSC papers were obtained in ‘Hypertext Markup Language (HTML)’ format. The search query consisted of the following two criteria. First, papers must contain the full phrase “thermally activated delayed fluorescence” in its text. Secondly, the paper titles must not contain “complex” or “polymer” so that most of the papers would report on molecular TADF materials.

### Document pre-processing

The thus obtained HTML and XML files contain structured contents of the articles where markup tags were used to label information within the tags, such as authors and digital object identifiers (DOIs). However, tagging conventions vary across publishers and journals. ChemDataExtractor provides template scrapers for Elsevier and RSC journals to convert HTML and XML contents into standard structured ChemDataExtractor “Document” objects. The scraping removes information that is irrelevant to property text mining and segment articles by content type. The “Document” object can subsequently be parsed using a universal text-mining pipeline for journals.

### Natural language processing (NLP)

ChemDataExtractor v2.1 embeds cutting-edge NLP techniques, which are specifically tailored to mine textual data about chemical materials for applications in physics and chemistry. The toolkit performs bespoke segmentation of documents from the scientific domain, and thence sentence-level tokenization, part-of-speech (POS) tagging, and chemical-named entity recognition (CNER); the results of which form the features that are used in later stages of data extraction. The CNER capabilities of ChemDataExtractor 2.1 are enabled by a language model that is based on bidirectional encoder representations from transformers (BERT); this simultaneously affords state-of-the-art organic and inorganic compound-name recognition. The NLP module of ChemDataExtractor 2.1 is mainly unchanged, except for some special treatment for the detection of abbreviations for organic molecules.

Organic molecules often have long systematic names to encode their structural and compositional information. Abbreviations are used extensively in the field of TADF, and the correct recognition of abbreviations is crucial for attributing the correct parent compound to an associated property. The default abbreviation detector in ChemDataExtractor was adapted from the work of Schwartz and Hearst^22^. It requires that the letters in an abbreviation appear in the same order within its corresponding full name. This rule generally covers well the case of scientific acronyms, but it is often insufficient for organic molecules, such as e.g., 4,5-Bis(carbazol-9-yl)-1,2-dicyanobenzene (2CzPN). Therefore, the rule was replaced by a new one for this work such that an abbreviation is detected when a chemical name tagged by the CNER system is preceded or followed by a shorter entity with correct brackets or equality signs.

### Information extraction

**Fig. 1:**
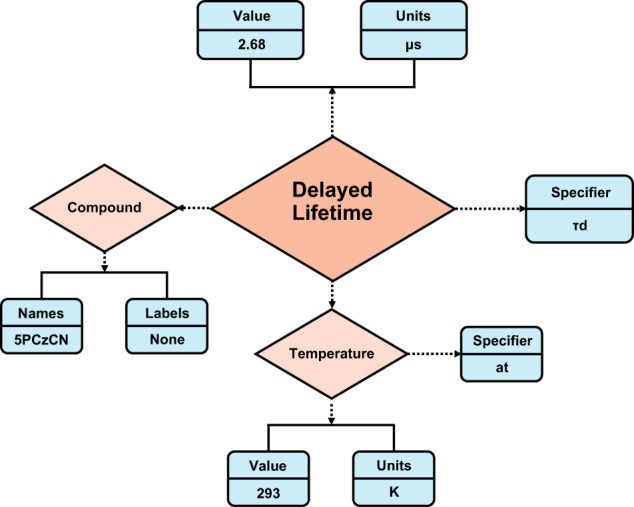
Example of an extracted ChemDataExtractor v2.1 model for TADF-based research. The ontology shows the values, units, chemical compound, specifier, and the temperature at measurement that are associated with a Delayed Lifetime record.

ChemDataExtractor 2.1 allows an ontology called “model” to be defined for each desired property and the extraction pipeline attempts to populate it. A user can define the dimension of a property within the model and ChemDataExtractor will automatically generate parse expressions for the property. The user also needs to define the specifier of the property, e.g., *τ*_D_ for delayed lifetimes. An example of a populated ontology for delayed lifetime is shown in Fig. 1, where the property, its specifier, compound, and other user-specified fields have been found in a paper and extracted.

ChemDataExtractor 2.1 provides template parsers for both sentences and tables in order to extract information and populate models. The sentence template parsers take the plain text of a sentence, POS tags and CNER tags of its tokens, and compare these against hand-crafted semantic relationships for a match. These relationships have already been implemented within ChemDataExtractor and no further user input is required. The table parsers require an extra step of concatenating a table cell and its corresponding headers with the separator ‘‘🙃🙃🙃🙃” into a sentence-like string. During cell parsing, the value of a property, its units, corresponding compound, and other required fields are sought within this string on a first-come-first-served basis. A model will be extracted once a match has been found. The metadata and citation parts of a document are not parsed for property records.

Three special table parsers were built to cover specific edge cases of TADF properties. The first was designed for Δ*E*_ST_, which is sometimes given in the same column as singlet and triplet energies under a table header *E*_S_/*E*_T_/Δ*E*_ST_. The second was developed for fractional PLQY values in tables. A third parser was developed for delayed lifetimes that are reported in the same table cells as photoluminescence quantum yield, emission wavelength, or prompt lifetime.

### *ThemeCompound* model

ChemDataExtractor 2.1 has a new feature that allows information found elsewhere to be merged into a parent model. However, there is no inter-sentence relationship written in rule-based parsers and the merging is done in the order of inter-record distance. This leads to a common source of false positive property records where reagents, substrates, and compounds of comparison are mistaken as the compound possessing the property.

Here, we introduced the *ThemeCompound* model for ChemDataExtractor, which has background knowledge of the scientific domain being text-mined, such that it looks at only the focal compounds that are being introduced in a paper. The generation of this knowledge is realised automatically through the ChemDataExtractor v2.1.

**Fig. 2:**
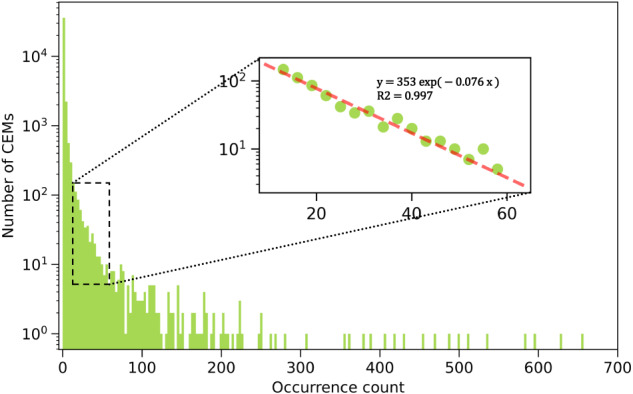
Chemical entity mention (CEM) occurrence distribution for the ~87k CEMs found by ChemDataExtractor v2.1 when 1200 RSC papers of TADF research were extracted using the built-in ‘Compound’ model. The x-axis represents the number of papers in which a specific CEM occurs, while the y-axis shows the number of CEMs with a given occurrence count in log-scale. There is a highly linear region in the distribution as plotted in the inner box, implying that the number of CEMs drops exponentially with the occurrence count in this intermediate regime.

The procedure behind the *ThemeCompound* model is now described. First, 1200 RSC papers were extracted using the built-in Compound model yielding ~ 87,000 records about chemical entity mentions (CEMs). Names of the CEMS were standardized by removing unnecessary white spaces. From these records, a CEM-occurrence-frequency table can be constructed. A CEM is only counted once for multiple occurrences within the same paper. The distribution is plotted in Fig. 2. Here, 81.9% of the CEMs appeared in only one document and the number of compounds drops dramatically from one to ten occurrences. This is followed by a highly exponential decay from ten to nearly a hundred occurrences (*cf*., inset). The top occurring compound is “nitrogen” (found in 655 documents), which is a gas commonly used to generate an inert atmosphere, while the second-most occurring compound is “toluene” (found in 627 documents), which is a common solvent.

It is necessary to filter out these commonly used compounds. For that purpose, we chose to blocklist compounds that appear in seven or more out of the 1200 documents, which accounts for 3.5% of all compounds. The blocklist was further enriched by name variations of the same compound. Finally, humanly compiled rules were applied on top of the blocklist, as shown in Table 1. *ThemeCompound* will inevitably blocklist some well-known TADF molecules, such as DMAC-TRZ. However, as the properties of these molecules are readily available in many sources, they were added to our database during post-processing.

**Table 1:** Additional human compiled blocklisting rules enforced in the *ThemeCompound* model.

Field	Blocklisting Rules
Names	Separator ‘‘🙃🙃🙃🙃’’.
	DOI strings of current document.
	Chemical entity mentions (CEMs) followed by “-based”, part, segment and alike.
	CEMs ending in “o” (amino), “yl” (phenyl) and alike prefixes.
Labels	Strings of dates, electronic states, units.
	Numbers followed by % or wt %.
	Strings longer than 4 characters.

### Forward-looking resolution of compounds

The new BERT-based CNER system deployed in ChemDataExtractor 2.1 significantly improves the detection of organic compound names and abbreviations using language models. Nevertheless, the same CEM can either be recognized or not, depending on its surrounding text. More consistent CNER capabilities were realised by implementing a forward-looking dependency resolution into ChemDataExtractor 2.1. This software version has been modified for this work so that it can remember the names and labels of *ThemeCompound*s once they have been detected in a document by the CNER system, and these memories are reset when a document-extraction process concludes. Furthermore, compound names and labels under table headers, such as “Compounds” and “Emitters” are also treated automatically as *ThemeCompound*s.

The generative behaviour of this compound-memorising mechanism and its discriminative behaviour from the filtering process forms a competing pair that affords better performance in both precision and recall for the extraction of correct CEMs that are associated to TADF properties.

### Classification of experimental Δ*E*_ST_ records

Δ*E*_ST_ values can be estimated from either density-functional theory (DFT) calculations or experimental techniques, such as spectroscopy and transition rates measurements. Theoretically and experimentally sourced data each accounted for approximately a half of all Δ*E*_ST_ records. Therefore, we decided to assign Δ*E*_ST_ records with a field “is_experimental” to inform database users of the distinction.

The classification was done with a classifier finetuned from MatSciBERT^23^. For a data record, the input data for classification consists of the heading of the section, the first sentence of the paragraph, and the sentence containing the data record. A training set of 208 human labelled records was collected. The classifier was constructed by adding a linear binary classification layer on top of the MatSciBERT model, and the model was finetuned on the training set for 5 epochs. The classification was done for all Δ*E*_ST_ records originating from text and for table records that are linked to text records. Standalone table records cannot be confidently classified because of the lack of contextual information.

### Data post-processing and cleaning

Data records were simplified to remove redundant nesting layers. Metadata of the document, where a record was found, was added as the “metadata” attribute of the record. For each property, data records lying outside a physically reasonable range were removed. These ranges were set to be 300 nm < *λ*_EM_ < 1600nm, 0 % ≤ PLQY ≤ 100 %, −1.5 eV < Δ*E*_ST_ < 1.5 eV, and 100 ns < *τ*_D_ < 10 s. For the singlet-triplet energy splitting values, there are a few papers that report them in units of kJ/mol and these were converted into eVs during the cleaning stage of our operational pipeline. Finally, for the temperature attribute of the delayed lifetime records, room-temperature flags were replaced by temperature models of 293 K, and conflicts with extracted temperature models were resolved.

Then, we computed the SMILES string for all property records that contain a compound with a full and valid chemical name according to the International Union of Pure and Applied Chemistry (IUPAC). This procedure was carried out using PyOPSIN, a Python wrapper for the JAVA package Open Parser for Systematic IUPAC nomenclature (OPSIN)^24^. Molecules that have fewer than 24 carbons and possess fewer than five aromatic rings were removed.

## Data Records

**Fig. 3:**
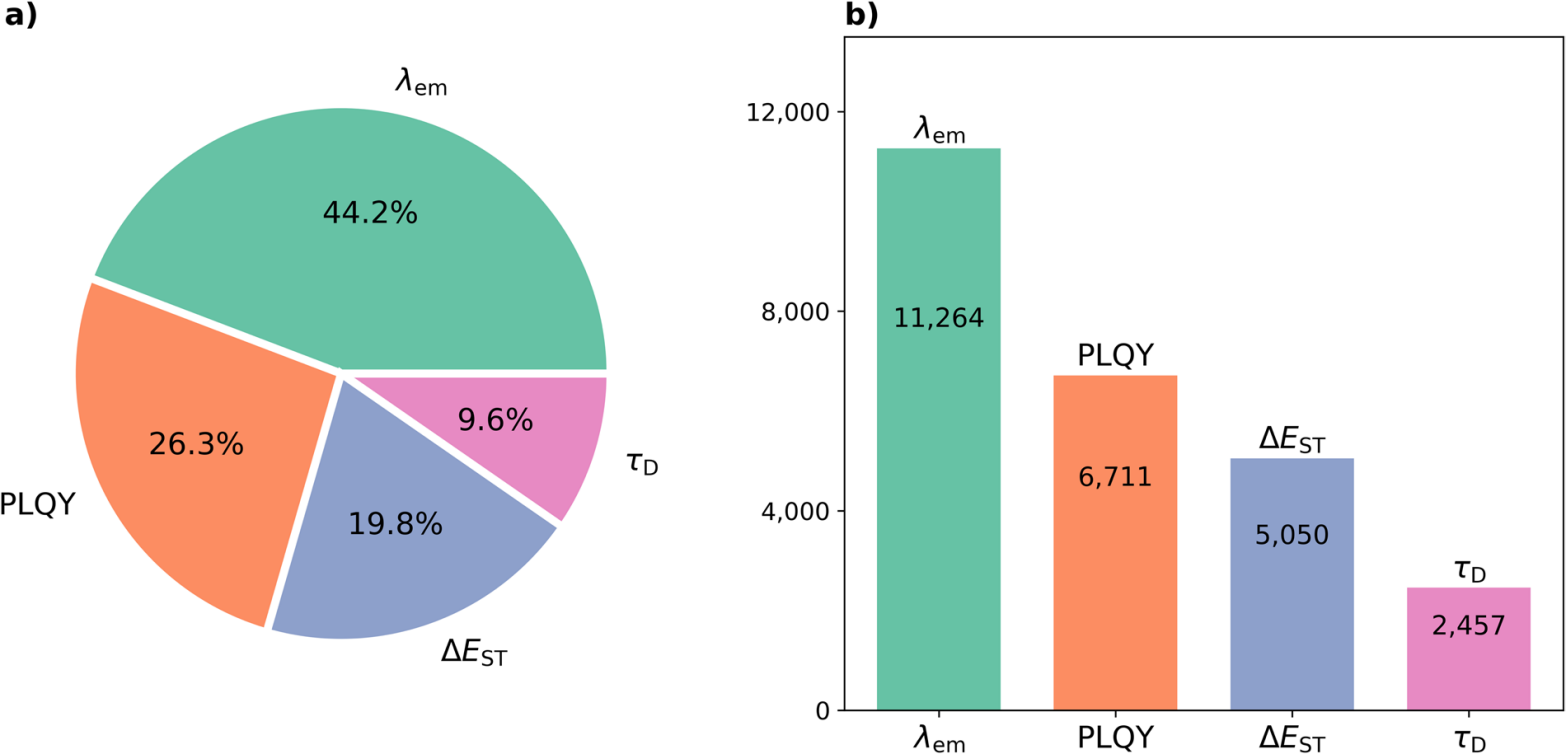
Composition of the four extracted TADF-related properties in the master database, namely, λ_EM_, PLQY, Δ*E*_ST_, τ_D_, in descending order of the number of data records found for each property. Pie chart **(a)** shows the percentages accounted by the four properties, while the bar chart **(b)** shows the number of extracted data records of each property.

A master database and a subsidiary database are provided, each containing data records from all four target properties. The master database contains 25,482 records and 9,820 unique compounds (Fig. 3). The subsidiary database contains 5,349 records where a SMILES string could be associated with their compounds, among which 1,318 unique chemical structures were found. Both databases have been made publicly available at *FigShare*^25^ in CSV, JSON, and MongoDB formats.

All records in the master database share a common base structure. This includes a ‘model_name’ field that distinguishes among the four properties. The ‘compound’ field includes the name and labels of the compound associated with the record. The ‘value’ and ‘units’ fields contain a list of values and the unit string of the found property record, respectively. In addition, we defined a standard unit for each of the four properties; these are nanometre (nm), percent (%), electron volt (eV), and microsecond (μs) for *λ*_EM_, PLQYs, Δ*E*_ST_, and *τ*_D_, respectively. Accordingly, a list of standard values in their standard units was added for each property record as the ‘standard_value’ field. The ‘record _method’ field contains the name of the parser yielding the record. The metadata field contains a dictionary that details the published citation, e.g., authors, journal name, and year of publication. In the subsidiary database, property records have the same structure as in the master database, except that an additional SMILES field is nested under the compound dictionary.

For the PLQY model, there are two additional fields of a) ‘phase’ that reports the physical state of the chemical and the host material, and b) ‘atmosphere’ which states if the measurement was conducted in nitrogen or air. The *λ*_EL_ model also has the field of ‘phase’. Delayed lifetime records have an extra field of ‘temperature’ that gives the temperature at the point of its measurement. Δ*E*_ST_ records have an extra field of ‘is_experimental’ to denote if the record was estimated from experiments or theoretical calculations in the original paper.

## Technical validation

Precision and recall metrics were adopted to evaluate the quality of the data-extraction pipeline^13^. Precision refers to the percentage of correct records among all extracted records; recall is the percentage of correct records among all existing records in the corpus where text-mining was performed. These are given by

$${\rm{P}}{\rm{r}}{\rm{e}}{\rm{c}}{\rm{i}}{\rm{s}}{\rm{i}}{\rm{o}}{\rm{n}}=\frac{{\rm{T}}{\rm{P}}}{{\rm{T}}{\rm{P}}+{\rm{F}}{\rm{P}}},$$

$${\rm{R}}{\rm{e}}{\rm{c}}{\rm{a}}{\rm{l}}{\rm{l}}=\frac{{\rm{T}}{\rm{P}}}{{\rm{T}}{\rm{P}}+{\rm{F}}{\rm{N}}},$$

$${\rm{F}} \mbox{-} {\rm{score}}=2\cdot \frac{{\rm{precision}}\ast {\rm{recall}}}{{\rm{precision}}+{\rm{recall}}}.$$

**Fig. 4:**
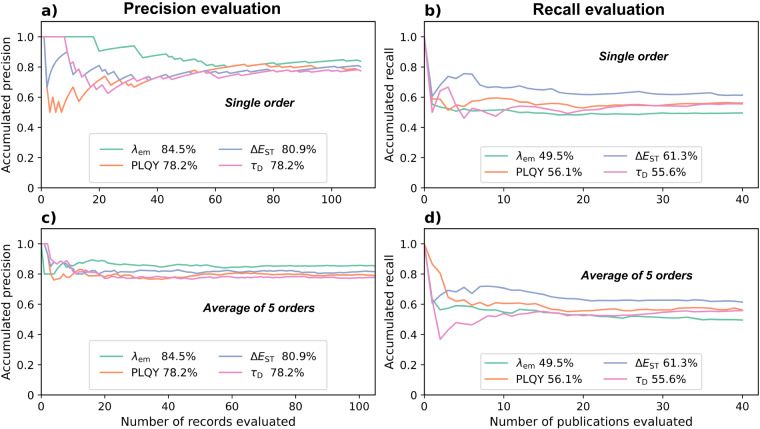
Evaluations of precision and recall for the four extracted properties for TADF-based materials. Plots **(a)** and **(b)** show the accumulative precision and recall, respectively, for a particular accumulating order of the randomly selected evaluation set. Plots **(c)** and **(d)** display the accumulative precisions and recalls, respectively, averaged over five accumulating orders. Hence, non-coincidental convergence is confirmed.

TP, FP, and FN are the number of true positive, false positive, and false negative records, respectively. A record is considered to be true positive if it contains: (i) the correct property with both the value and the unit, (ii) either the correct compound name or the compound label if they are mentioned in the same paragraph or table as the property phrase, (iii) the correct temperature value and unit if the record is a delayed lifetime record. In order to obtain reliable metrics for the database, 110 randomly selected records for each property were evaluated to determine the precision. Moreover, 40 publications that yielded at least two records were randomly selected for the evaluation of the recall. The accumulative metrics, as a function of the number of records or publications, are plotted in Fig. 4a,b for all four property models; the precision and recall values are printed next to their legends. The metrics were observed to converge with fluctuations of <0.5% within the evaluated samples. The shape of the convergence curves naturally depends on the order in which the records or publications are evaluated. To confirm non-coincident convergence, we computed the metrics in five random accumulating orders and the average trends are plotted in Fig. 4c,d. Faster convergence is seen for both precision and recall of all four properties. Therefore, we concluded that the convergence is not coincidental.

For the subsidiary database that contains only the records with SMILES strings, 43 randomly chosen records were evaluated and a precision of 90.7% was obtained. For the ‘is_experimental’ flag in Δ*E*_ST_ records, 980 out of 1283 Δ*E*_ST_ records in the subsidiary database were flagged while 2620 out of 5050 records in the master database were flagged. Furthermore, 64 randomly selected records were evaluated, and 92.2% overall flag precision was obtained. We also analysed proportions of techniques used in Δ*E*_ST_ estimations to ensure data consistency. After evaluating 100 randomly selected records, 68 records were found to have originated from experiments. 97% of experimental records were extracted from the difference in onsets of fluorescence and phosphorescence spectra while the rest were estimated with transition rates.

The precision and recall values for all four property extractions are listed in Table 2. The final row shows the average data-extraction performance across the four properties. The mean precision is 82% with a standard error of 2%; the mean recall is 56% with a standard error of 3%; the mean F-score is 0.65 with a standard error of 0.02. The low standard error across the four extracted properties indicates high stability of our database auto-generation pipeline.

**Table 2:** Precision, recall, and F-score of the four TADF-related properties extracted from literature.

	Precision [%]	Recall [%]	F-score	Number of records
*λ*_EM_	84.5	49.5	0.624	11,264
PLQY	78.2	56.1	0.653	6,711
Δ*E*_*ST*_	80.9	61.3	0.697	5,050
*τ*_*D*_	78.2	55.6	0.650	2,457
Mean	82(2)	56(3)	0.65(2)	N/A

Text mining of emission wavelengths yielded the lowest recall (49.5%), which is primarily due to the circumstance that language used by authors of papers when reporting emission wavelengths is often much more flexible and versatile than that for the other three properties. Therefore, it is more difficult to cover all embedded records in publications. Moreover, complicated syntactic structures reduced the precision of extracting the compound bearing the emission-wavelength property. Conversely, the singlet-triplet energy splitting values in publications are mostly denoted symbolically (Δ*E*_ST_ or minor variations thereof), which explains the highest recall of 61.3%.

By comparing common FPs, precision is lowered mainly by the failure to extract compound names or labels. A large majority of these errors are associated with a failure of CNER while extracting properties from tables, where there is often a lack of semantic context, especially for review articles where compounds are not explicitly defined and tables with multiple-layer nesting structures are adopted.

**Fig. 5:**
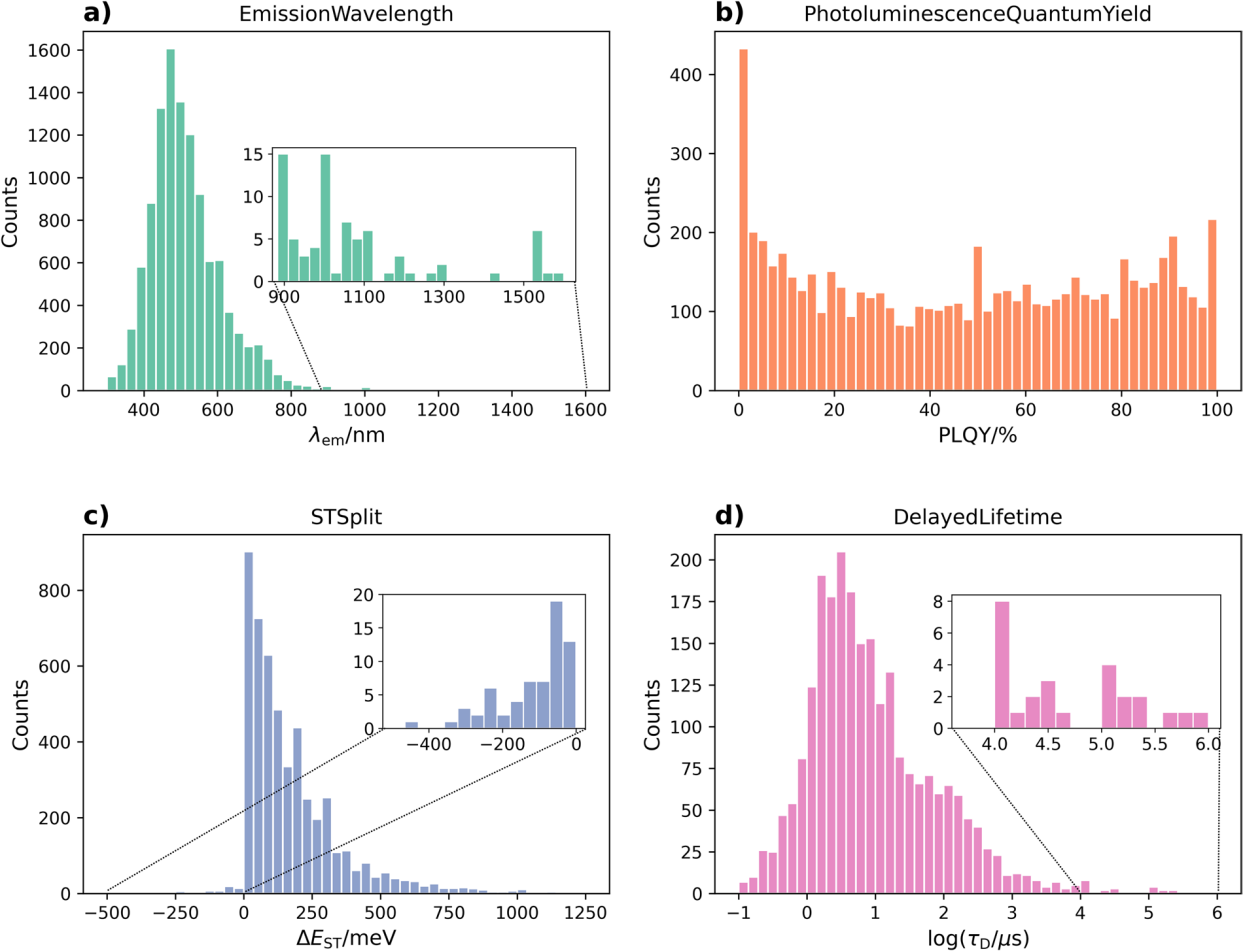
Distribution of the four properties that are extracted for TADF-based materials. **(a)** Distribution of λ_EM_ values displays a dominant single peak in the visible spectrum, with minor peaks in the IR range, which are related to bio-imaging applications. **(b)** Distributions of PLQY values are mostly uniform from 0% to 100%; the spike near zero is attributed to both actual vanishing PLQY values and incorrect units in some tables. **(c)** ΔE_ST_ values are mostly positive due to electron exchange effects and the number of records drops rapidly with increasing value. Negative ΔE_ST_ values have also been achieved and can be explained by double excitations^28,29^. **(d)** Distribution of τ_D_ values is plotted with x-axis in log-scale due to their large spread across orders of magnitude. Most data records lie within the microsecond range, while few records lie in the millisecond range, most of which are associated with phosphorescent materials with restricted RISC.

The distributions of the four extracted property values are plotted in Fig. 5. The emission-wavelength value distribution matches the expectation that it has a single peak with most data lying within the visible spectrum. Within the near-infrared (NIR) range, there are two dominant peaks at 900 nm and 1000 nm referring to CAT-1^26^ and TPAAZ^27^ and their close variants, respectively. Most research with data records in this range were driven by the interest of advancing bio-imaging applications, especially for brain-related imaging^27^.

The density of data records for the PLQY value distribution is roughly uniform from 0% to 100%. Spikes are seen at tens of percents, which are attributed to the approximative rounding-to-ten description of PLQY values. A similar pattern has been observed for the extracted data of the thermoelectric figure of merit, *ZT*^13^ and for various battery device properties^17^. The spike at 0% is particularly prominent, which includes PLQY values between 0% and 2%. By inspecting these data records, there exist ~70 records with vanishing PLQYs, which are stated within the literature in approximate forms such as “ < 1%” and “~0%”. In addition, there are several tables in papers, wherein PLQY headers declare that the properties are given as percentages, while the actual cell values have really been normalised and are given out of unity^15^. Unfortunately, these reporting errors can only be identified by manual comparison to mentions of the same property in the text of the paper on a case-by-case basis. The second prominent peak at 100% was attributed to the theoretical possibility of TADF materials in potentially achieving a complete PLQY value of unity, which is claimed in the introduction of most TADF publications.

For Δ*E*_ST_ data records, their distribution of values peaks at near-zero, and suddenly drops to vanishing levels when crossing from the positive to the negative x-axis. This can be rationalized by considering that singlet states are generally more energetic than corresponding triplet states due to the exchange interactions. However, inverted singlet-triplet gaps have been theoretically and experimentally demonstrated by considering contributions from double excitations^28,29^.

In the histogram for the delayed lifetime, the majority of the records lie in the microsecond scale with a spread from hundreds of nanoseconds to hundreds of microseconds. The horizontal axis is in log-scale to account for the relatively large spread in orders of magnitude. There are sparse records in the millisecond to sub-second range. Most of these are reporting triplet state lifetimes in systems with limited RISC, where the main decay pathway is phosphorescence with a spin-change, which leads to prolonged lifetimes.

## Usage Notes

The master database and the subsidiary database have been made available in CSV, JSON, and MongoDB (BSON) formats, which can be downloaded from *Figshare*^25^. The master database and subsidiary database are as described in the *Data Records Section*. One can interact with these databases using standard database languages, such as e.g., Structured Query Language (SQL) and MongoDB Query language; these database formats are also widely supported by many general programming languages, such as Python, R, and MATLAB.

## Data Availability

The code used to generate the databases in this work can be found at https://github.com/Dingyun-Huang/chemdataextractorTADF. The repository contains ChemDataExtractor v2.1 which has been modified for text-mining TADF properties; iPython notebooks that demonstrate an example data-extraction pipeline and data-cleaning and post-processing are also provided.
